# Prevalence of Helminthiasis Among African Elephants in Tsavo and Laikipia-Samburu Ecosystems, Kenya

**DOI:** 10.3390/vetsci12121161

**Published:** 2025-12-04

**Authors:** Sophia K. Mogaka, Evans M. Mwangi, Folorunso O. Fasina, Wilkinson W. Mutahi

**Affiliations:** 1 Department of Biology, University of Nairobi, P.O. Box 30197, Nairobi 00100, Kenya; emmwangi@uonbi.ac.ke (E.M.M.); mutahiwthuku@gmail.com (W.W.M.); 2 Food and Agriculture Organization of the United Nations, 00153 Rome, Italy; 3 Department of Veterinary Tropical Diseases, University of Pretoria, Onderstepoort, Pretoria 0110, South Africa

**Keywords:** African elephant, gastrointestinal parasites, helminth prevalence, Kenya, *Murshidia*, parasite ecology, Laikipia, Tsavo

## Abstract

African elephants maintain a delicate balance between high intestinal helminth numbers and symptoms of poor health. However, calves and weaners face survival threats from damage caused by helminth infections and infestations during periods of starvation. Using coprological evaluations, we compared helminth infection patterns and selected risk factors in elephants from Tsavo and the Laikipia-Samburu ecosystem (LSE). Eggs and adult worms were identified and classified into genera using morphological and morphometric characteristics. Overall prevalence rates were high at 95.6% and 98.5% for Tsavo and Laikipia-Samburu, respectively, and 97.1% in total. Using the chi-squared test of independence, nematode prevalence rates (97.1%) were significantly different from trematodes (32.6%) (χ2(1) = 248.84, *p* < 0.001). Prevalence rates did not differ across age classes in the two populations. Using the Generalized Linear Model, sex had an independent significant effect on the observed mean worm burdens (F(1) = 5.842, *p* = 0.016, CI 95%), but neither age nor location did. However, the interaction had a significant effect on mean eggs per gram (EPGs) observed between age and location (F(1) = 14.043, *p* < 0.0005, CI 95%) and between age and sex (F(1) = 4.858, *p* = 0.028, CI 95%). Some of the identified helminth species may potentially lead to life-threatening gut mucosa hemorrhaging and ulcerations, particularly during stressful conditions, although the animals were not clinically evaluated.

## Simple Summary

This study investigated gut worms (helminths) in African elephants from two major Kenyan ecosystems: Tsavo (TENP) and Laikipia-Samburu (LSE). Researchers found that worm infection is extremely common, with over 95% of elephants in both areas carrying these parasites. While healthy elephants typically coexist with the worms without showing symptoms, the study identified a serious hidden threat. Specific worms found can cause life-threatening gut bleeding and ulcers, especially during stressful periods like drought or food shortage. The research also revealed that female elephants and those living in family herds have higher worm loads than males. This is likely due to their social behavior and the stress of caring for young. The study concludes that high parasite loads, combined with environmental pressures like habitat loss, are a significant risk to elephant health. This is particularly true for vulnerable calves, and wildlife managers should monitor this threat closely.

## 1. Introduction

Previous studies have evidenced the catastrophic effects that helminths may inflict upon the elephant host [1,2,3]. These include pathological lesions, hemorrhages, tissue necrosis, and even death [2,3,4]. Helminth infections also suppress the host’s immunity, stagnate growth, and decrease reproduction [5].

Due to a well-developed parasite-host equilibrium, the African elephant is usually asymptomatic for gastrointestinal (GI) parasites [6]. Heavy worm burdens, however, pose a serious threat to survival. They abrade the epithelial lining of the intestines, causing damage to the rich microvasculature and gaining access to use the host’s ingested nutrients, leading to nutritional deprivation, particularly in times of starvation.

Parasite infestations do not always lead to clinical symptoms. In most free-ranging populations, intestinal parasites are neglected, with a resultant threat to the health and, ultimately, survival of especially calves and weaners. Elsheikha and Obanda [7] hypothesized that disease is maintained at subclinical levels as a result of co-evolution between hosts and parasites, also cited more recently [6,8]. A parasite-host equilibrium has therefore been established [9]. In particular, helminths produce immune-evasion molecules that help maintain a balance between worm expulsion and virulence. However, clinical signs of disease may begin to show upon destabilization of the parasite-host equilibrium. This can occur due to factors such as concurrent infection, pregnancy and lactation, or adverse weather changes.

The most common helminths in African elephants are nematodes (*Murshidia* sp., *Khalilia* sp., and *Quilonia* sp.), followed by trematodes (*Protofasciola* sp., and *Fasciola* sp.). Intestinal coccidian infections are common, but have not been associated with any clinical symptoms [1]. In Kenya, the nematode *Grammocephalus clathratus*, the trematode *Protofasciola robusta,* and a number of unidentified adult worms were recovered from elephant carcasses during the drought of 2009, where 38 young animals aged between 5 and 8 years died in the Laikipia-Samburu ecosystem (LSE) [3]. Eleven of the carcasses revealed pathological lesions and hemorrhages that were linked to parasitism. This suggests that helminths could play a potentially important role in regulating wildlife populations.

The aim of this study was to investigate the prevalence of helminth infections and understand species diversity and intensity within two of the largest ecosystems inhabited by elephants in Kenya.

## 2. Materials and Methods

### 2.1. Study Area

**Figure 1:**
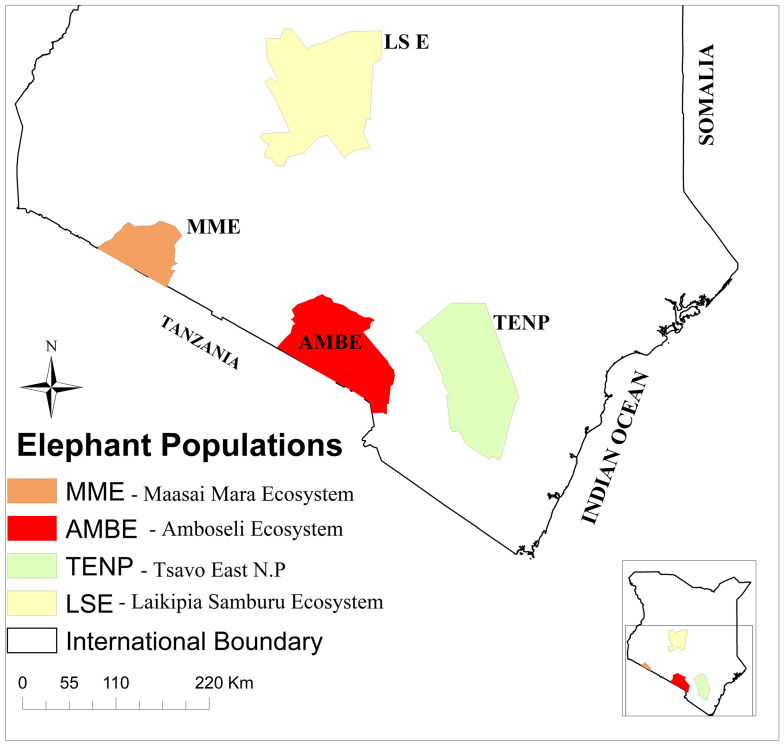
Partial map of Kenya showing elephant distribution in Kenya, including TENP and the LSE, our areas of study [4].

This study was undertaken in the Tsavo East National Park, the largest in Kenya and considered to be one of the largest in the world, with an approximate area of 13,747 km^2^, and the Laikipia-Samburu ecosystem, which covers approximately 25,000 km^2^, located in the central heartland of the country (Figure 1).

Rivers Galana, Tiva, and Voi traverse the park, along which is a narrow riverine forest and thicket dominated by *Acacia elatior*, *Hyphanae compressa,* and *Suaeda monoica*. The northern part is more densely vegetated, predominantly *Acacia commiphora*, while the southern part has been opened up over the years by fire and elephants [10]. Common shrubs here include the genera *Premna*, *Bauhinia,* and *Sericocomopsis*, and scattered trees such as *Delonix elata*, *Melia volkensii,* and baobab (*Adansonia digitata*). The area experiences two unpredictable rain seasons, between April and May, and November and December. The average annual rainfall ranges between 300 mm and 600 mm [11]. Threatened wildlife in this park includes the critically endangered black rhino (*Diceros bicornis*) and hirola (*Damaliscus hunteri*), the vulnerable elephants (*Loxodonta africana africana*), cheetah (*Acinonyx jubatus*), and leopard (*Panthera pardus*). Other species in the park include African buffalo (*Syncer caffer*), lions (*Panthera leo*), several antelope species, including the fringe-eared oryx (*Oryx beisa callotis*), waterbucks (*Kobus ellipsiprymnus*), and lesser kudu (*Tragelaphus imberbis*), and hundreds of bird species, to mention a few [11]. Based on the Kenya Wildlife Service [4], this ecosystem is estimated to hold around 7727 elephants.

The Laikipia-Samburu ecosystem is semi-arid, with a similar bimodal rainfall pattern, with an annual average of around 300 mm in the north and 700 mm in the south. The ecosystem is characterized by hills, plateaus, and rough terrain with six major land use types: national reserves, state-protected forest reserves, communal pastoral areas, community conservancies, private ranches, and settlements under subsistence production. Wildlife found in this ecosystem include the introduced, near-threatened, Northern white rhino (*Celatotherium simum cottoni*), the black rhino, the endangered reticulated giraffe (*Giraffa camelopardalis reticulata*), the Grevy’s zebra (*Equus grevyi*), the vulnerable Somali ostrich (*Struthio molybdophanes*), elephants, cheetahs (*Acinonyx jubatus*), and leopards (*P. pardus*); the beisa oryx (*Oryx beisa*), the gerenuk (*Litocranius walleri*), several other antelope species, buffaloes (*S. caffer*), lions (*P. leo*) and hundreds of bird species. The elephant population in this area at the time of this study was estimated at 7166 [12].

### 2.2. Sample Collection

Freshly voided fecal samples were collected between June and October 2019, following the methods of Baines et al. [1] throughout the week during this whole period. Once spotted, elephant herds or lone males were observed until they had defecated and moved off to a safe distance. We targeted watering holes in the afternoon and opportunistically in the mornings and evenings.

Samples were collected using 50 mL and 100 mL fecal pots, gloves, wooden splints, and 10% formalin as a preservative. Approximately 50 g of fecal material was picked from different boluses from the same animal, including some from the top, center, and bottom of the bolus. The wooden splints were used to stir the fecal sample with the 10% formalin to ensure homogenization and uniformity in preservation. The fecal pots were then sealed and stored. At the same time, demographic variables (age, gender, and social group) were collected and matched with each individual sample. All the samples were collected before the rains in both locations and were transported to the veterinary laboratory at the KWS headquarters for analysis.

A total of 273 individual elephants were examined for intestinal helminths. Out of these, 137 (50.2%) individuals were from TENP and 136 (49.8%) individuals from the LSE.

### 2.3. Sample Processing and Analysis

Parasitological examination and qualitative analysis were performed using centrifugal Sheather’s sugar flotation, water sedimentation, while quantitative analysis used McMaster egg counting. Fecal material was sorted through to identify the presence of adult worms.

#### 2.3.1. Centrifugal Flotation Method

The flotation solution was prepared by mixing 454 g of table sugar with 355 mL of distilled water to prepare a Sheather’s sugar solution, with a specific gravity of 1.27.

Helminth eggs were concentrated using the centrifugal Sheather’s sugar flotation technique as described by King’ori et al. [11]. Approximately 3 g of fecal sample was processed, and the resulting preparation was examined under a microscope (X400) (MICROTEC IS300, ISCapture. Ink, Exeter, UK). Micrographs were taken, and dimensions of helminth ova were measured using the ISCapture micro-imaging software version (MICROTEC IS300, ISCapture. Ink, Exeter, UK).

#### 2.3.2. Sedimentation Method

A modified water sedimentation was used, where 3 g of the fecal sample was weighed and mixed with 45 mL of tap water [11]. The mixture was strained, and the filtrate was transferred to a 50 mL centrifuge tube. The filtrate was left to sediment, and the supernatant was gently decanted. The sediment was re-suspended and decanted 2–3 times to achieve a clear suspension. An amount of 50 µL of the sediment is pipetted onto a glass slide and covered with a coverslip for observation. Image processing follows the same process as that of the flotation method.

#### 2.3.3. McMaster Method

To quantify the burden of helminth infestation, a modified McMaster method adopted from Foreyt [13] was used, using Sheather’s sugar as the flotation fluid. It follows the same procedure as the centrifugal Sheather’s sugar flotation described above, until the point where the sediment is mixed with the Sheather’s sugar solution to form an upper meniscus. No cover slip was placed at this point; instead, the mixture in the 15 mL centrifuge tubes was left to stand to allow helminth eggs to float to the surface. A pipette was used to transfer suspension from the surface to a McMaster slide, filling both chambers. The slide was placed on a microscope (MICROTEC IS300, ISCapture. Ink, Exeter, UK) and eggs inside the chambers counted under ×10 magnification.

For both quantitative and qualitative analysis, 137 fecal samples from TENP and 136 fecal samples from the LSE were analyzed in each case.

#### 2.3.4. Adult Worm Identification

We carried out manual sorting of fecal samples collected from TENP and the LSE to look for adult worms present in the dung. Adult worm processing was conducted following the modified method of McLean et al. [14]. Briefly, worms found were placed in clean sample bottles containing 10% formalin. The worms were later placed on a slide and cleared using glycerol. A coverslip was placed on top of the slide, and the sample was left to sit for 1 week to achieve clearing before observation [3]. After a week, the slide was placed on the microscope (MICROTEC IS300, ISCapture. Ink, Exeter, UK) for observation under the X100 magnification, and images of anterior and posterior regions were recorded using ISCapture micro-imaging software version (MICROTEC IS300, ISCapture. Ink, Exeter, UK). Whole worms were observed using a dissecting microscope (Leica EZ4D), and images were captured using the supporting software LAS EZ (Leica Microsystems, Deerfield, Illinois, USA). To identify the genera present, we focused on studying the morphology of the anterior and posterior ends of identified worms, and compared these with standard published works [15,16,17].

Identification to the genus level was based on comparison of egg measurements taken from samples and published egg dimensions of helminths affecting African elephants [2,9,17]. Egg measurements were used to identify species by comparing them to known standards.

### 2.4. Data Analysis

Flotation and sedimentation data were used to calculate the prevalence of infection for comparison by location, sex, and age. The resulting count data on disease presence or absence were analyzed to determine prevalence across the areas of study, social groups, and age. Differences were tested for significance using chi square test of independence at 95% CI, using the IBM SPSS version 20 software (IBM, Armonk, USA).

To examine the effect of the interaction of location, age, social group, and sex on EPG, we utilized the Generalized Linear Model (GLM) with a fitted Poisson distribution function on the IBM SPSS version 20 software.

## 3. Results

We collected samples from social (male, 34.8%, and family, 65.2%) and age-class (adult, 58.6%; sub-adult, 24.9%; and juvenile, 16.5%) groupings (Table 1), with an overall prevalence of nematode infections being 97.1% for Kenya (Table 2). Elephants from TENP and the LSE were infected by a wide range of nematodes, mostly strongyles and a few trematodes. Overall, location-specific prevalence rates were not significantly different between TENP (95.6%) and the LSE (98.5%) (χ2(1) = 2.03, *p* > 0.05) (Table 2). The family social group in the LSE exhibited prevalence rates that were significantly different from the male social group (χ2(1) = 7.17, *p* < 0.05), while there were no significant differences observed in the elephant social groups in TENP (χ2(1) = 0.93, *p* > 0.05) (Table 3). Across ages, there were no significant differences observed either: TENP (χ2(2) = 1.54, *p* > 0.05) and the LSE (χ2(2) = 1.75, *p* > 0.05) (Table 4). The results indicated that, in general, rates of helminth infection and the egg loads (EPG) were not statistically different between elephants in TENP and in the LSE (F(1) = 0.25, *p* > 0.05). Sex had a significant effect on mean worm burden (F(1) = 5.84, *p* < 0.05), with females exhibiting higher EPGs than males.

### 3.1. Intestinal Parasites in Elephants from TENP and LSE Areas

#### Egg Morphology and Morphometry and Adult Worm Identification

**Figure 2:**
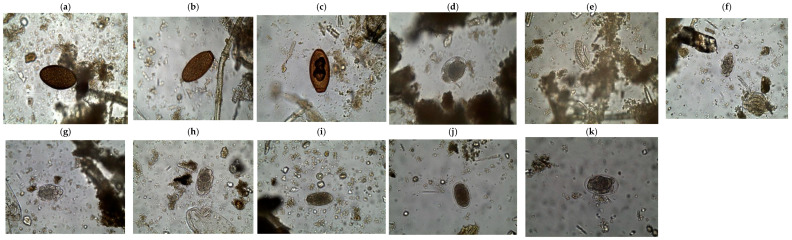
(**a**–**k**) Photomicrographs of eggs from nematodes and trematodes of four genera: (**a**) *Fasciola* sp. (99.47 × 52.42 µm), (**b**,**c**) are different sizes of *Protofasciola* sp.: (**b**) (98.39 × 50.28 µm), (**c**) (102.53 × 53.02 µm): (**d**–**f**) are different sizes of *Murshidia* sp. (71.2 × 50.46 µm) (**e**) (72.27 × 39.07 µm) (**f**) (65.13 × 37.22 µm); (**g**–**i**) are different sizes of *Quilonia* sp.; (**g**) (80.02 × 45.79 µm) (**h**) (82.08 × 43.99 µm) (**i**) (76.55 × 38.63 µm); (**j**) *Grammocephalus* sp. (70.36 × 43.06 µm); (**k**) *Khalilia* sp. (83.52 × 49.85 µm).

A total of 273 elephants were sampled for this analysis, 137 (50.2%) from TENP and 136 from the LSE (49.8%). Populations from the sampled areas are infected by nematodes whose eggs were of a typical strongyle-type morphology, previously described by Baines et al. [1]. Some nematode genera identified from eggs and their subsequent morphometry are shown in Figure 2a–k. They included those of *Quilonia* sp. (80–90 × 40–55 μm), *Murshidia* sp. (70–75 × 35–50 μm), *Grammocephalus* sp. (65–75 × 40–50 μm), and *Khalilia* sp. (80–92 × 44–60 μm). Trematode species identified had similar lengths to those of *Protofasciola* sp. (84–104 × 56–64 μm) and *Fasciola* sp. The eggs of *Fasciola hepatica* were easily distinguishable from those of the other trematodes due to the absence of an operculum, which is distinct in *Protofasciola robusta* and *Brumptia bicaudata* species [9].

**Figure 3:**
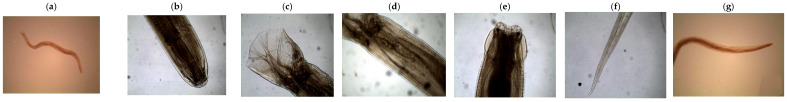

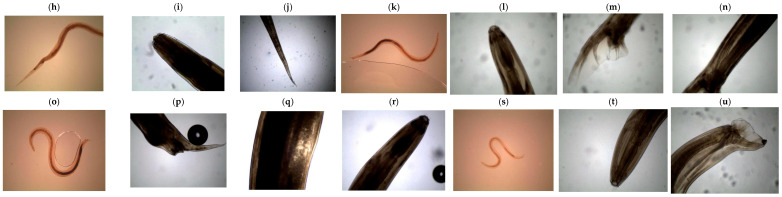
(**a**–**u**) Photomicrographs of adult nematode worms isolated from fecal samples. *Quilonia* sp. male: (**a**) whole worm X8, (**b**) anterior/head region X100, (**c**) tail showing copulatory bursa X100, (**d**) tail region showing spicules X100), *Quilonia* sp. female: (**e**) anterior region/head X100, (**f**) tail X100, *Murshidia* sp. female: whole worm X8: (**g**) anterior, (**h**) posterior, (**i**) anterior region/head X100, (**j**) tail region X100, *Murshidia* sp. male: (**k**) whole worm X8: (**l**) anterior/head region X100, (**m**) tail region with copulatory bursa X100, (**n**) tail region with arrow showing spiculon X100, *Murshidia* sp. female: (**o**) worm X8, (**p**) tail region X100, (**q**) eggs in gravid female X100, (**r**) anterior/head region X100, *Murshidia* sp. male: (**s**) whole worm X8: (**t**) anterior/head region X100, (**u**) tail region showing copulatory bursa and spicules X100.

No adult worms were recovered from the TENP samples. However, from the LSE samples, 29 worms were recovered from 8 out of the 136 samples collected. Of the worms recovered, 26 (89.7%) were identified up to genus level. Using keys and descriptions provided by Anderson et al., [15], Monnig, [16] and Van Der Westhuysen, [17], infections from *Quilonia* sp. (Figure 3a–f) and *Murshidia* sp. (Figure 3g–u) were identified. The morphology of the anterior and posterior regions of the worms were found most useful for identification.

### 3.2. Comparison of Helminth Occurrence in TENP and the LSE

#### Comparison of Prevalence

Based on social grouping, individuals sampled from male social groups were 34.8%, while those sampled from family social groups were 65.2%. Adult elephants sampled in this study were 58.6% of the total, of which 87 individuals were from TENP and 73 from the LSE. Sub-adults were 24.9%, with 30 individuals from TENP and 38 from the LSE. Lastly, the total number of juveniles sampled was 16.5%, with 20 individuals from TENP and 25 from the LSE (Table 1).

The prevalence rate established from the flotation method was 93%, while that obtained from sedimentation was 97.1%; this difference proved to be statistically significant (χ2(1) = 4.72, *p* < 0.05) (Table 2).

There was no significant difference in prevalence rates based on location (χ2 (1) = 2.03, *p* > 0.05). The prevalence of trematodes (32.6%) differed from that of nematodes (97.1%) (χ2(1) = 248.84, *p* < 0.001). Elephants from TENP (39.4%) had a trematode prevalence rate that differed from that observed from the population in the LSE (25.7%) (χ2(1) = 5.81, *p* < 0.05) (Table 2).

There was no significant difference in observed prevalence rates in the male and family social groups of the elephant populations of TENP (χ2 (1) = 0.93, *p* = 0.335). In the LSE, however, the family social group recorded a prevalence rate (100%) that differed from that of the male social group (93.3%) (χ2(1) = 4.715, *p* = 0.007). There was no significant difference in trematode prevalence based on social groups from both locations: TENP (χ2(1) = 3.55, *p* = 0.06) and the LSE (χ2(1) = 1.66, *p* = 0.198) (Table 3).

There is no significant difference in overall helminth prevalence rate observed between adults, sub-adults, and juveniles in both TENP (χ2(2) = 1.54, *p* = 0.462) and the LSE (χ2(2) = 1.75, *p* = 0.416). Trematode prevalence based on age also shows no significant differences in both TENP (χ2(2) = 1.4, *p* = 0.495) and the LSE (χ2(2) = 0.12, *p* = 0.943) (Table 4).

### 3.3. Comparison of Worm Burden

Generalized Linear Modeling at a 95% confidence level was used using IBM SPSS Statistics 20 to determine the effect of age, sex, and location on the worm burden. Based on this model, it was possible to determine the effect of location, age, and sex on the mean worm burden observed. Results obtained from the General Linear Model showed that age alone (F(1) = 0.789, *p* = 0.375, CI 95%) and location alone (F(1) = 0.247, *p* = 0.620, CI 95%) had no significant effect on mean worm burdens observed. Sex had a significant effect on mean worm burden (F(1) = 5.842, *p* = 0.016, CI 95%), with females exhibiting higher EPGs than males.

Based on location alone, though the difference between them was not statistically significant, adult elephants in TENP exhibited the highest EPG means, as indicated in Table 5, followed by sub-adults, with juveniles recording the least mean EPG. In the LSE, on the other hand, adult elephants recorded the least mean EPG, followed by juveniles and sub-adults recording the highest mean EPG, as shown in Table 5. Adults in TENP are more likely to experience higher intensity of worm burdens as compared to adult elephants in the LSE. However, juvenile and sub-adult elephants in the LSE are more likely to harbor more helminths than those in TENP.

Infection intensity in adult females was higher compared to adult males, while sub-adult males and females seem to have similar levels of infection intensity. Mean EPG in adult females is higher than that of sub-adults, while adult males have lower egg burdens as compared to sub-adults. It proved difficult to sex elephant calves while collecting samples in the field; hence, the missing comparison of sex and age with regard to this group.

## 4. Discussion

Our study revealed that African elephants exhibit a high prevalence of helminth infections with varied patterns between elephant populations in the TENP and LSE. It should be known that elephants’ habitat loss and fragmentation have led to elephants’ splitting into many sub-populations as a result of movement restriction. King’ori et al. [11] have suggested that these elephant sub-populations are likely to suffer different rates of parasite infestation, and in our study, we reported intestinal parasites’ prevalence of 95.6% and 98.5% in elephants from TENP and the LSE, respectively. Other workers have also reported high prevalence rates in Kenya, including 87.5% by Elsheikha et al. [18] and 97.5% by King’ori et al. [11]. These studies established that, irrespective of age, social group, or location, African elephants are highly susceptible to helminths, especially nematodes.

In our study, more nematode infections (97.1%) than trematode infections (32.6%) were observed. Similarly, King’ori et al. [11] in Kenya, and Baines et al. [1] in the Okavango Delta, have reported more nematodes (97.5% and 73%) than trematodes (39.1% and 26%), respectively. The complex life cycle of the trematode may have accounted for the lower prevalence rates, which include the necessity of an intermediate host (aquatic snails), whose presence is largely determined by the presence of a permanent water source. Where these special conditions are not met, trematode infection may be suboptimal compared to nematodes, which undergo a more direct lifecycle [18]. However, during the seasons with an abundance of water, elephants do enjoy lengthy periods of time in the water bodies to cool their body temperatures on hot days. Such behavior may serve as a risk factor and increase trematode infection. In addition to ecological factors, the lower trematode prevalence in our findings may also be influenced by methodological limitations. While sedimentation allowed efficient recovery of nematode eggs, trematode eggs are fewer, heavier, and often embedded within fecal debris, making them harder to detect consistently. Sedimentation involves multiple washing and decanting steps, during which heavier trematode eggs can remain trapped within fecal particles or be inadvertently discarded together with supernatant. Thus, the lower trematode prevalence reported here may partially reflect diagnostic limitations rather than true biological absence.

Apart from the differences in the life cycles of nematodes and trematodes, the abundance and distribution of aquatic snails within the ecosystem serve as risk factors for trematode prevalence. Factors that support aquatic snail distribution include physicochemical water quality (water temperature, dissolved oxygen, ions and salts in the water, depth, and availability of food), predation, and others, all of which affect snail species distribution, with resultant effect on trematode prevalence [19]. Although we determined that elephants from TENP recorded higher trematode prevalence than those from the LSE, King’ori et al. [11] have observed a different result where elephants from the LSE recorded higher trematode prevalence than those from TENP. A full-scale malacological survey of wildlife habitats in Kenya, covering all seasons, would therefore be useful in shedding light on trematode distribution and prevalence in animals in the wild. We hypothesized that location rather than social group influences trematode prevalence.

Elephants’ grazing habits encourage re-infection with nematodes. Grazing areas and the general savannah are characterized by scattered fecal material from elephants and other wild animal species that are found in these areas. In the dry season, most adult elephants preferred to feed on shrubs and trees due to the unavailability of grass. Where grass was found, it was scanty, dry, and very close to the ground. Elephants would use their trunk to collect grass, and use their feet to remove dirt and soil from the grass before ingesting. Such feeding habits in contaminated areas will lead to inadvertent ingestion of helminth ova, creating conducive conditions for infection and re-infection, a situation that may account for the high nematode prevalence in elephant populations.

The presence of *Protofasciola robusta* and *Fasciola hepatica* can be attributed to the presence of marshes, swamps, streams, and even possible watering holes that provide suitable environments for the intermediate snail hosts. Since elephants feed in marshy areas, there is a possibility of ingesting metacercariae, with consequent infection with these parasites. *P. robusta* has been isolated from the duodenum and distal entrance of the bile ducts and small intestines of elephants [2,3], and the parasite has been associated with hemorrhage, intestinal tissue damage, and calf fatality [3]. *Fasciola hepatica* adults have been found occupying the elephant’s bile ducts and can lead to anorexia, constipation, jaundice, anemia, and ultimately death. Fowler and Mikota [9] have explained that chronic infection could lead to obstruction of bile ducts, elevation of intrahepatic blood pressure, hypoproteinemia, hemorrhage, and death.

We detected the eggs of elephant hookworm, *Grammocephalus clathratus,* in this study. The adults of these worms inhabit the host’s liver and bile ducts, causing hemorrhages and tissue damage in these organs [3]. Heavy infestation in the bile ducts by these three species can occlude the bile ducts and cause eventual death. Similarly, *Murshidia* and *Quilonia* species, whose pathology has not been well defined, were occasionally found in the large intestines and sometimes in the small intestines [2]. While healthy elephants are asymptomatic to helminth infections, conditions like starvation and nutrient deprivation by helminths can cause pathological lesions on the intestinal mucosa [3], and in aggravated helminthosis, elephants’ survival fitness may be greatly compromised.

In this study, we utilized opportunistic non-invasive methods to obtain adult worms that were excreted together with feces. Because helminth species from the same host are subject to extreme variations [17], uncertainties in species classification may exist. We utilized multiple methods to classify the *Murshidia* sp. and *Quilonia* sp. up to the genus level [15,16,17]. These two species have earlier been found highly concentrated in the caecum and colon of elephant hosts by Condy [2]. The possible pathological effects of these two species in elephants have been discussed earlier [14,20,21]. Their eggs have also been identified by King’ori et al. [10] from Kenyan elephants across various populations.

We recorded higher mean EPGs in family social groups than in the male social groups, an indication that the social structure in elephant populations does have an effect on the intensity of helminth infections [11]. Family groups tend to associate in larger herds as compared to bachelor herds and lone bulls. This association in large herds creates an environment of re-infection, as they tend to feed for long in the same areas, and they defecate in these areas as they feed, as explained earlier. In addition, infection intensity in the social groups is a factor of the foraging dynamics found in elephant social groups. Family herds rarely move far from water sources in dry seasons. This is because family herds consist of calves and sub-adults who may not move as fast as adults and are at higher risk of predation and mortality due to exhaustion. Lone bulls and bachelor herds, on the other are not held back and can therefore travel further distances, in search of water and even better food in times of drought, with less risk [22]. Thus, in the dry season, when family herds are utilizing dwindling and diminishing resources, the adult bulls are able to acquire better feed and water by traveling further. This, therefore, means that family herds will undergo nutritive and hydric stress more, leading to an increase in the intensity of helminth infections due to lowered immunity and thus record higher EPGs as compared to their male counterparts, as observed in this study. Mean EPGs recorded reveal similar patterns of infection intensity, whereby female elephant hosts are more parasitized than male hosts [1,5,11,17]. However, some studies that have looked at the effect of sex on helminth infections in elephants confirmed opposite results [21]. In our case, mean EPGs recorded in female elephants (707) and male elephants (556) were an indication of relatively moderate levels of infection intensity by helminth parasites.

Overall, the patterns observed in the study showed that elephants from TENP have a higher mean EPG (623.06 ± 653.798) compared to elephants from the LSE (589.34 ± 589.237) (*p* > 0.05), and adults in TENP had significantly higher EPGs compared to adults in the LSE. Perhaps the habitat range and resource distribution in the LSE and TENP played a role in this observation. TENP is a gazetted and fenced national park, covering approximately 12,000 km^2^. The park has one main source of permanent water, the Galana River, with seasonal sources including the Tiva and Voi rivers, Aruba Dam, scattered ponds, swamps, and watering holes [23]. In the dry seasons, elephant home ranges in TENP shrink considerably as water resources become scarce. The elephants retreat to areas along the Galana, Voi, and Tiva rivers to increase their chances of survival in the dry season [23]. The reduction in home range, therefore, increases chances of heavy parasite infestation, especially for family social groups, due to foraging in the same grasslands over a prolonged period of time. The LSE, on the other hand, covers a much larger area of 33,817 km^2^ [24]. The ecosystem has a wide range of habitats, associated with climatic gradients within the region: hot and dry lowlands in the north, cool wet highlands to the south, interrupted with rugged mountains and open landscapes. The ecosystem allows for mostly free movement of elephants between the different land uses due to the wildlife corridors maintained in these areas [24]. Elephant populations in the LSE, therefore, have access to a wider range of habitats as compared to those in TENP. During the dry season, elephants in the LSE expand their home range in search of water and food, as was observed in Samburu National Reserve during the conduct of this study. Elephant data from the Save the Elephants Foundation included elephant families that were residents of the reserve, migratory herds, and newcomers. This could explain the differences in mean egg burden observed between the two locations. We suggest that elephants in TENP experience more stress in dry periods due to a reduction in habitat range and water resources as compared to those in the LSE, and therefore experience a higher mean egg burden.

This study has made some significant new contributions that were not present in the study in the same area [11]. First, while King’ori et al. [11] relied entirely on egg morphology and statistical clustering (Gaussian finite mixture models) to infer the genera of helminths present, our study went a step further by manually sorting fecal samples and successfully recovering 29 adult worms from Laikipia-Samburu elephants. We used detailed morphological keys to identify them to the genus level (*Quilonia* and *Murshidia*). This provides concrete, physical evidence of the parasites present, moving beyond statistical inference. Secondly, while King’ori et al. [11] mentioned that parasites can impact health, our manuscript places a much stronger emphasis on the potential pathological consequences, by specifically naming and discussing the life-threatening damage caused by species like *Protofasciola robusta*, *Fasciola hepatica*, and *Grammocephalus clathratus* (e.g., gut hemorrhaging, bile duct obstruction, and liver damage). We explicitly link these to mortality events during droughts, making the health implications more urgent and tangible for wildlife managers. Thirdly, we recovered substantially higher mean egg counts (EPG) than those in King’ori et al. [11]. We also provided a more nuanced discussion on why family groups and females have higher worm burdens. Ours is not just to sociality, but specifically to their foraging dynamics and constrained movement due to calves, which forces them to graze in more contaminated areas, especially during dry seasons. Fourthly, we offered a detailed comparison of the habitat structure and elephant movement patterns between the fenced Tsavo and the more open Laikipia-Samburu ecosystem to explain the differences in EPG, particularly in adults. Fifthly, we specifically identified the “elephant hookworm,” *Grammocephalus clathratus*, from its eggs. This species is notable for residing in the liver and bile ducts and causing significant pathology. Its identification adds another layer of specific health risk that was not highlighted in the King’ori et al. paper [11].

Overall, this study provides significant novelties beyond previous work by (1) successfully recovering and morphologically identifying adult *Murshidia* and *Quilonia* nematodes from free-ranging elephants, and (2) offering concrete genus-level confirmation that moves beyond inferences from egg morphology alone. (3) It highlights specific, high-pathology species like *Grammocephalus clathratus* and links them to tangible health risks such as gut hemorrhaging and bile duct obstruction, particularly during stressful periods of droughts. (4) Furthermore, our analysis offers a nuanced explanation for higher worm burdens in females and family groups, tying it to constrained foraging mobility and resource stress, and contrasts how fenced versus open ecosystems (Tsavo vs. Laikipia-Samburu) shape parasite pressure.

Using egg measurements alone for the identification of species present presents a few challenges. It has been noted that egg measurements for a single species vary greatly across different elephant populations [11]. To overcome this downfall, it is important, where possible, to study larval stages and adult worm morphology to determine the species present. The most assured way, however, for species determination is through molecular characterization [14].

Whether host species variation exists was not studied in this work, but this knowledge is needed as part of the elephant ecological system and will provide insight into the parasite-host equilibrium. The patterns observed, including sex, age, and location, may affect mean worm burdens in elephant populations, but more studies are needed to understand the Kenyan ecosystems’ elephant worm burdens and establish ‘high/low worm burden’. This is useful for routine surveillance and health monitoring in wildlife (elephant) health. Ecosystem fragmentation increases pressure on animals (population, movement, and resources), which may even be more dire during drought, and these are worsened by high helminth prevalence, especially in calves. Finally, this work is subject to certain limitations: we are aware of potential underestimation of trematodes due to the diagnostic methods used, and the inability to molecularly confirm species or assess host genetic or immunological factors influencing burdens. Future studies utilizing more specific methods should take account of these limitations and build on our work.

## 5. Conclusions

Helminth parasite families/species identified in this study are potentially harmful to elephants, especially during stress. The high gastrointestinal parasite burdens should be considered during purposive translocation and during the period of migration, in order to minimize the risk of infections and outbreaks. This work should assist wildlife managers to stratify and monitor subpopulations that may be worst afflicted in times of adverse climatic events.

## Figures and Tables

**Table 1:** Distribution and classification of sampled individuals across age and social grouping.

Category	Group/Age Class	TENP (*n*)	LSE (*n*)	Total (*n*) %
Social grouping	Male groups	65	30	95 (34.8)
	Family groups	72	106	178 (65.2)
Age class	Adults	87	73	160 (58.6)
	Sub-adults	30	38	68 (24.9)
	Juveniles	20	25	45 (16.5)

**Table 2:** Prevalence of helminth infections in elephants from Tsavo East National Park and the Laikipia-Samburu ecosystem, Kenya.

Category	Variables	TENP	LSE	Total	χ2	*p*-Value
	Total Count (N)	137	136	273		
Nematode infection	Number infected (n)	131	134	265		
	Prevalence (%)	95.6	98.5	97.1	4.72	<0.05
Trematode infection	Number infected (n)	54	35	89		
	Prevalence (%)	39.4	25.7	32.6	6.47	<0.05
		χ2 = 71.63, *p* < 0.05	χ2 = 112.03, *p* < 0.05	χ2 = 90.80, *p* < 0.05		

TENP is Tsavo East National Park, and LSE is the Laikipia-Samburu ecosystem. All estimation was performed based on the sedimentation method.

**Table 3:** Prevalence of helminth infections based on elephant social groups in the Tsavo East National Park and Laikipia-Samburu ecosystem, Kenya.

Category	Variables	TENP	LSE	Total
Male social group	Count (N)	65	30	95
Nematode infections	Number infected (n)	61	28	89
	Prevalence (%)	93.9	93.3	93.7
Trematode infections	Number infected (n)	31	5	36
	Prevalence (%)	47.7	16.7	37.9
Family social group	Count (N)	72	106	178
Nematode infections	Number infected (n)	70	106	176
	Prevalence (%)	97.2	100	98.9
Trematode infections	Number infected (n)	23	30	53
	Prevalence (%)	31.9	28.3	29.8

TENP is Tsavo East National Park, and LSE is the Laikipia-Samburu ecosystem. All estimation was performed based on the sedimentation method.

**Table 4:** Prevalence of helminth infections based on elephant age groups in the Tsavo East National Park and Laikipia-Samburu ecosystem, Kenya.

Category	Variables	TENP	LSE	Total
Adult	Count (N)	87	73	160
Nematode infections	Number infected (n)	83	71	154
	Prevalence (%)	95.4	97.3	96.3
Trematode infections	Number infected (n)	34	18	52
	Prevalence (%)	39.1	24.7	32.5
Sub-adult	Count (N)	30	38	68
Nematode infections	Number infected (n)	28	38	66
	Prevalence (%)	93.3	100	97.1
Trematode infections	Number infected (n)	10	10	20
	Prevalence (%)	33.3	26.3	29.4
Juvenile	Count (N)	20	25	45
Nematode infections	Number infected (n)	20	25	45
	Prevalence (%)	100	100	100
Trematode infections	Number infected (n)	10	7	17
	Prevalence (%)	50	28	37.8

TENP is Tsavo East National Park, and LSE is the Laikipia-Samburu ecosystem. All estimation was performed based on the sedimentation method.

**Table 5:** Mean helminth burden (EPG feces) for different age groups, social groups, and sexes in African elephants from Tsavo East National Park and the Laikipia-Samburu ecosystem, Kenya.

Elephant Population	N	Mean EPG ± SD	Median EPG
TENP	137	623.06 ± 653.798	400
Family social group	72	630.68 ± 591.977	425
Male social group	65	624.59 ± 741.542	400
Adult	80	733.73 ± 737.709	500
Sub-adult	30	566.67 ± 545.567	375
Juvenile	20	280.45 ± 247.252	225
LSE	136	589.34 ± 589.237	400
Family social group	106	685.85 ± 623.727	525
Male social group	30	248.33 ± 230.996	150
Adult	73	469.29 ± 536.717	300
Sub-Adult	38	847.56 ± 648.648	750
Juvenile	25	502.00 ± 509.591	300
Total female EPG	101	706.89 ± 625.963	600
Total male EPG	127	555.12 ± 650.788	350
Total populations’ EPG	273	606.26 ± 621.558	400

EPGs = eggs per gram; TENP = Tsavo East National Park; LSE = Laikipia-Samburu ecosystem.

## Data Availability

The original data presented in the study are openly available in the Repository of the University of Nairobi, Kenya (https://erepository.uonbi.ac.ke/handle/11295/164593) (accessed on 16 September 2025).

## Abbreviations

The following abbreviations are used in this manuscript:

CI	Confidence interval
EPGs	eggs per gram
GI	gastro-intestinal
GLM	Generalized Linear Model
IBM	International Business Machines Corporation
KWS	Kenya Wildlife Service
LSE	Laikipia-Samburu ecosystem
SPSS	Statistical Package for the Social Sciences
TENP	Tsavo East National Park
